# Is Systematic Biopsy Mandatory in All MRI-Guided Fusion Prostate Biopsies? A Machine Learning Prediction Model

**DOI:** 10.3390/cancers18030517

**Published:** 2026-02-04

**Authors:** Omer Longo, Gil Raviv, Miki Haifler

**Affiliations:** 1 Faculty of Medical and Health Sciences, Tel Aviv University, Tel Aviv 6997801, Israel; omeremmal@mail.tau.ac.il; 2 Department of Urology, Meir Medical Center, Kfar Saba 4428164, Israel; 3 Department of Urology, Sheba Medical Center, Ramat Gan 5265601, Israel; gil.raviv@sheba.health.gov.il

**Keywords:** prostate, cancer, fusion, systematic, biopsy, machine learning

## Abstract

Objectives: To develop a prediction model able to accurately predict which patients will harbor higher risk prostate cancer in the systematic biopsy template compared to the targeted biopsy during MRI/US fusion biopsy. Methods: We included patients who underwent fusion biopsy. Clinical and radiographic variables were collected from patients’ records. The outcome of the model was higher risk prostate cancer in the systematic compared with targeted biopsies. An extreme gradient boosting model was trained and tested. We evaluated variable importance and clinical benefit. Results: Five hundred and twenty-nine patients were included. Eighty-two (15.5%) patients had higher risk prostate cancer in the systematic biopsies. The area under the ROC curve and negative predictive value were 0.82 and 0.92, respectively. The four most important features for outcome prediction were prostate volume, PSAD, patient’s age, and PSA. The decision curve showed increased clinical benefit of our model at threshold probabilities of 0–0.5. Limitations include the retrospective design of the study and the lack of external validation of the model. Conclusions: We developed a prediction model able to accurately predict which patients must undergo systematic and targeted biopsy. This prediction model has the potential to help in the decision whether to perform SB and thus may lower the adverse event rate while keeping a high detection rate.

## Simple Summary

Prostate biopsies usually include targeted samples from magnetic resonance imaging lesions plus systematic samples across the prostate. Systematic sampling can detect extra cancers, but it adds needle cores and may increase bleeding, infection, discomfort, and costs, and often does not change treatment because decisions depend on the highest-risk cancer found. The authors analyzed 529 men who underwent both methods and built a machine-learning model from routine pre-biopsy information, including age, prostate size, prostate-specific antigen, and prostate-specific antigen density, to predict when systematic cores would contain a higher-risk cancer than targeted cores. In internal testing, the model showed good discrimination (area under the curve of 0.82) and a high negative predictive value (0.92); if systematic cores were omitted when the model predicted no benefit, clinically significant cancer would have been missed in 0.7% of patients. This approach could personalize biopsy schemes and reduce unnecessary cores, but it needs prospective validation.

## 1. Introduction

Prostate cancer (PC) is the most common non-cutaneous malignancy in the male population, with an estimated 300,000 new cases and 66,000 deaths in 2024 in the United States [1]. PC diagnosis is achieved by the combination of prostate MRI and targeted MRI/US fusion biopsy (FB) [2]. Tissue is obtained from suspected lesions seen on the MRI (targeted biopsy, TB) and systematic biopsy (SB) of the entire prostate [3]. The added value of SB on top of TB was 5.2% and 2.3% in the biopsy naïve and prior negative biopsy populations [4,5]. In a prospective clinical trial assessing the diagnostic accuracy of TB and SB, 14% of clinically significant PC’s (csPC) were found only in the SB samples. Furthermore, if no SB were performed, 5.2% of csPC would have been missed [5]. Clearly, there is a role for SB in the diagnostic pathway of PC. However, since treatment of PC is decided based on the highest risk PC found in the biopsies, lower risk csPC in the SB compared to TB does not change treatment decisions; it is redundant but may increase the risk of procedural complications (bleeding, infection). In the FUTURE randomized trial, each additional biopsy core increased the risk of adverse event by 11% [6]. We hypothesize that the patients harboring higher risk csPC in the SB compared with TB can be accurately identified based on clinical and radiologic factors, thereby individualizing the biopsy scheme, which in turn may lower complication rates and healthcare costs.

The present study aimed to develop and internally validate a machine-learning prediction model, able to accurately predict which patients will harbor a higher risk of PC in the SB compared to the TB. Only these patients benefit from SB on top of TB.

## 2. Materials and Methods

After IRB approval and waiver of informed consent at Sheba and Assuta medical centers, we reviewed the medical records of patients who underwent transrectal software-based FB from 2020 to 2024, with the Uronav (Philips, Amsterdam, The Netherlands) and Koelis Trinity (Koelis, Meylan, Auvergne-Rhône-Alpes, France) systems, respectively. All patients (regardless of prior biopsy history) with PIRADS 3–5 lesions on MRI were referred to FB, performed by two urologists (M.H, G.R) experienced with more than 300 FBs and using both systems regularly. In all cases, 2–3 cores were obtained from each region of interest (ROI) seen on the MRI, and 8–12 systematic cores. We included in our study all patients who underwent FB at the two included institutions. We excluded patients lacking FB pathology or MRI reports and PSA values. The following variables were collected from the electronic medical record: patient’s age, PSA, prostate volume assessed by MRI, prostate region of the ROI (peripheral zone (PZ), other), PIRADS score v2.1, clinical T stage (rectal exam and MRI), highest Gleason score (GS) and grade group (GG) in the TB and SB separately. These are readily available pre-biopsy variables that are used in decision-making in the PC space. PSA density (PSAD) was calculated as follows:

PSAD = (PSA[ng/mL])/(Prostate Volume[cc])

As we wish to predict biopsy results before the biopsy, we included only pre-biopsy predictors (age, prostate volume, PSA, PSAD, cT stage, Highest PIRADS, DRE, and location of the highest PIRADS).

### Statistical Considerations

Continuous and categorical variables were described with median (inter-quartile range, IQR) and proportion (%), respectively. Continuous variables were compared with the Mann–Whitney U rank test. Categorical variables were compared with Fisher’s exact test. Specifically, the highest PIRADS and cT stage were defined as ordinal variables. The entire cohort was randomly split into training (70%) and test sets (30%). The outcome of the model was the presence of higher risk PC in the SB compared with TB (SB dominant, SBD):

SBD = SB_GG > TB_GG

where SB_GG and TB_GG are the highest Gleason groups in the SB and TB, respectively.

The PC risk was determined by the GG on a Likert scale. Extreme Gradient Boosting (XGBoost, XG) is a machine learning algorithm for regression and classification that uses an ensemble of weak prediction models, typically decision trees. As an ensemble tree model, XG uses multiple iterative gradient boosters to construct a strong classification system. As a non-linear classifier, XG can capture non-linear relations between features, which logistic regression cannot. XG is fast, provides high performance, and has been used extensively in the medical field [7,8]. We used XG with 10-fold cross-validation to train the model with the training set. An automated grid search was performed to find the optimal hyperparameters of the model. Sample size calculations for the proposed model were implemented in R using the “pmsampsize” package [9]. Under the assumptions of a binary outcome (SBD), an AUC of 0.9 and a maximum of eight predictive variables, a minimum of 227 cases is needed. Model performance was assessed with the receiver–operating characteristic (ROC) curve. Shapley Additive Explanation (SHAP) scores were used to identify each variable’s influence on the final prediction [10]. The model’s prediction for a particular patient can be expressed as the sum of the SHAP values for each feature plus a baseline value. This allows for a clear understanding of how each feature contributes to the prediction. Positive SHAP values mean the feature value increases the model-derived outcome probability, while negative values decrease it. For each feature, the mean SHAP value across all patients was calculated. We transformed the mean SHAP values into percent of influence on prediction probability (relative variable importance) as follows:

Relative variable importance = (variable mean SHAP value)/(∑_(all variables)▒〖mean SHAP value〗)*100

Calibration of the model probabilities was assessed with the Brier score calculated on the test set [11]. The clinical benefit of the model was assessed with decision curve analysis [12]. Missing data were analyzed and imputed by multiple imputations via the chained equation method [13]. The proportion of missing data is reported in Supplementary Table S1. Five imputed datasets were generated using an iterative fully conditional specification approach. For each imputation, 50 iterations were performed to ensure algorithmic convergence. Variables were imputed using predictive mean matching. A fixed random seed was used to ensure reproducibility, and pooled estimates were obtained using Rubin’s rules. Statistical analyses were performed with R v.3.6.1: R Foundation for Statistical Computing (Vienna, Austria). Multiple comparisons were accounted for by using the false discovery rate [14]. All tests were 2-sided, with significance considered at *p* < 0.05. All results reporting is in accordance with the TRIPOD statement for prediction model development and validation [15].

## 3. Results

Five hundred and twenty-nine patients were included in this study (269 at Sheba Medical Center and 260 at Assuta Medical Center), out of which 82 (15.5%) were SBD. The patients’ clinical data are presented in Table 1.

SBD patients were significantly older (74 vs. 66 years, *p* < 0.001) and had larger prostate volume (48 vs. 58 cc, *p* < 0.001). There were no significant differences in the number of lesions, maximal PIRADS score, histologic region, PSA or PSAD, and clinical T stage between the SB or TB dominant groups. There were no differences in any of the features between the training and test sets (Table S1).

**Figure 1:**
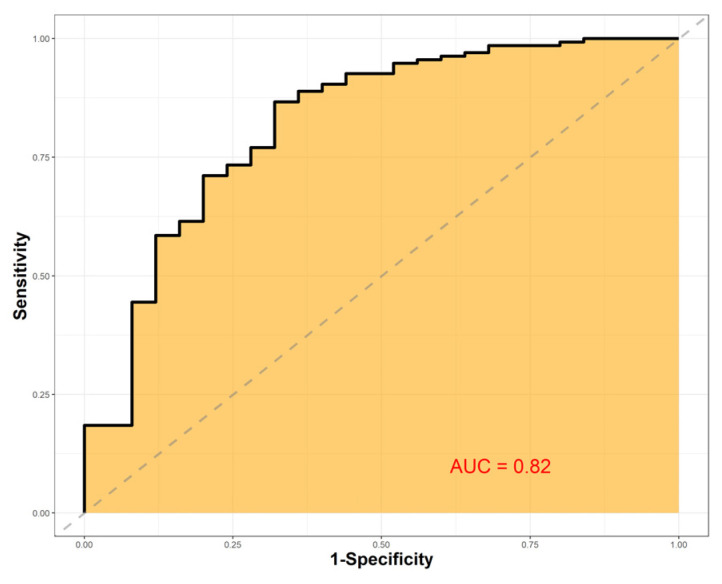
The proposed model demonstrates favorable characteristics with an AUC of 0.82 and a negative predictive value of 0.92.

We examined the performance of the trained model on the test set and found an AUC of 0.82 (0.79, 0.85) (Figure 1). With a threshold probability of 50%, seventeen (3.2%) cases were SBD but were classified by the model as TBD (false negatives, FN). Only in four (0.7%) FN cases, csPC was found in the SB and was absent in the TB. With this threshold, 130 (24.9%) SB’s would be omitted without any missed PC. The negative predictive value (NPV) of our model was 0.92. The precision-recall AUC was 0.7 (0.67, 0.74). Finally, the sensitivity, specificity, positive predictive value, and balanced accuracy were 0.2, 0.98, 0.71, and 0.6, respectively.

**Figure 2:**
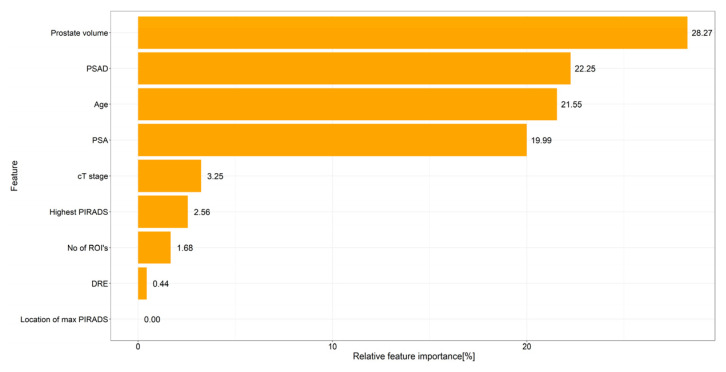
Relative feature importance as assessed by SHAP values. The model was mostly influenced by prostate volume, PSAD, patient’s age, and PSA (with relative importance of 28.3%, 22.25%, 21.5% and 20%, respectively).

The 4 most important features were prostate volume, PSAD, patient’s age, and PSA (with 28.3%, 22.25%, 21.5%, and 20% relative importance, respectively (Figure 2)).

**Figure 3:**
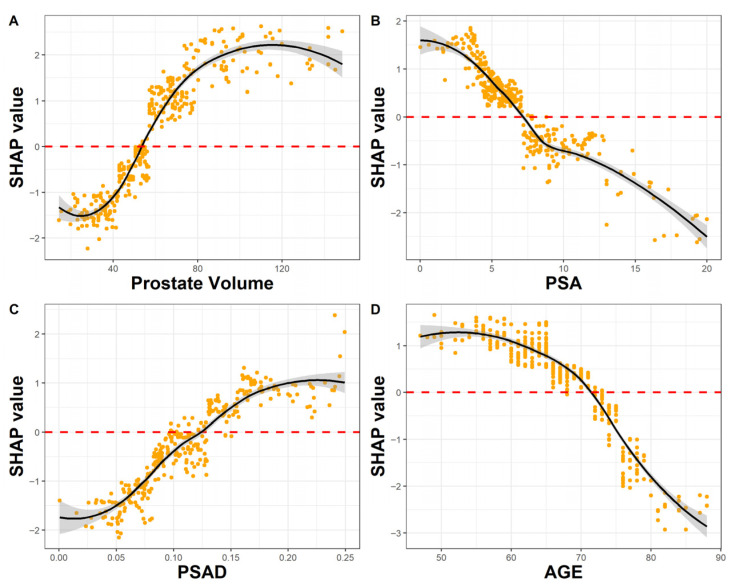
Dependence plots of the features with the highest relative importance. Yellow dots represent individual SHAP values; the black line indicates a smoothed trend, and the shaded grey area represents the 95% confidence interval. The red dashed line denotes a SHAP value of zero. Feature values with positive SHAP values increase the probability of SBD. (**A**) Prostates larger than 50 cc, (**B**) PSA < 7.5, (**C**) PSAD > 0.12, and (**D**) patients younger than 75 years all increase the probability of SBD.

Figure 3 shows the influence of different feature values on the final prediction of the model. Higher SHAP values indicate a higher model-derived probability of SBD. For instance, larger prostates produce higher probabilities of SBD. Prostates smaller than 50 cc lowered the probability of SBD (SHAP values from 0 to −2), while prostates larger than 50 cc increased it (SHAP values from 0 to 2.5). PSA values also influenced the model’s prediction, with values lower than 7.5 increasing SBD probability while higher values decreasing it. PSAD had a strong influence on the model’s prediction despite the inclusion of PSA and prostate volume in the model’s features. PSAD lower than 0.125 decreases SBD probability, while higher values increase it. Age has an inverse effect on SBD probability. Patients older than 70 years have a decreased probability of SBD.

**Figure 4:**
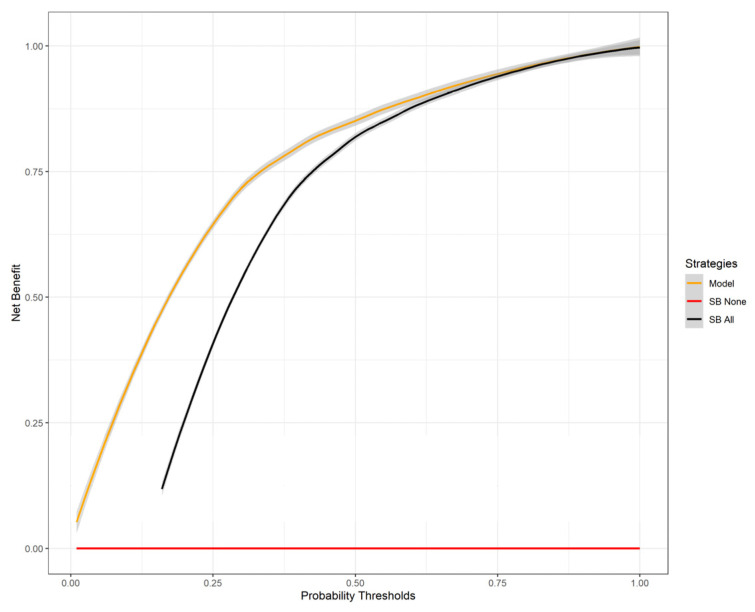
Decision curve of the proposed model. For threshold probabilities lower than 50%, the model provides a higher net benefit compared with the systematic biopsy of all patients strategy. If the urologist believes the chance of SBD is lower than using our model has clinical benefits.

The decision curve showed increased clinical benefit of our model at threshold probabilities of 0–0.5. In this range, there is a clinical benefit in using our model compared to performing SB in all patients or in no patient (Figure 4).

The Brier score of our model was 0.1, representing very good calibration between the model-derived probabilities of SBD and the actual prevalence of SBD in the test set.

## 4. Discussion

Approximately two million prostate biopsies are performed annually in the US and Europe, which makes it one of the most common urologic procedures performed [16]. Complications after these procedures pose significant health and cost-related issues and must be minimized. Common complications after prostate biopsies include urinary, rectal, and sperm bleeding (up to 84%), urinary retention (up to 11%), infections (15%), and hospitalization (6.9%) [16]. The association of the number of biopsy cores and complications is well understood. Pepe et al. performed a retrospective analysis of 4000 patients undergoing transperineal prostate biopsy and found an association between the number of cores and complications. The rate of acute urinary retention, hematospermia, and hematuria rose from 2.5%, 6.5%, and 6.8% with six cores to 11.1%, 30.4%, and 10.4% with >24 cores, respectively [17]. Reducing the number of biopsy cores may reduce the complication rates and health care costs while improving patients’ quality of life. However, lowering the number of cores may lower the cancer detection rates. In the “MRI-first” prospective trial, the csPC detection rates were 30%, 32%, 37% for SB, TB, and SB + TB, respectively [5]. In a retrospective study comparing the results of SB, TB, and SB + TB to radical prostatectomy specimens, Ahdoot et al. showed that omitting SB would lead to 7.7% decrease in csPC detection rate [18]. This finding might be explained by different mechanisms, such as csPC presence in the SB territory, but also by sampling the TB territory during the SB. Thus, Positive SB does not necessarily mean that SB was mandatory. Some balance must be struck between the high detection and complication rates. Since the current treatment paradigm is based on the highest risk PC detected in all cores, we looked to find those patients whose SB has a higher risk PC compared to the TB by using common pre-biopsy variables. In these patients, the SB is mandatory, while it can be omitted in all other patients. Our model provided accurate predictions of included patients with an AUC of 0.82. The FN rate of our model was low (3.2%). In these patients, SB would have been omitted, and a higher Gleason group in the SB would have been missed. Out of these patients, only 4 (0.7%) csPC would have been missed by performing only TB, which is an extremely low FN rate for a clinical prediction model. The NPV of our model was remarkably high (0.92), giving confidence to omitting SB in patients thought to be TBD by the model. On the other hand, the accuracy and sensitivity of our model are low (0.6 and 0.2). This highlights the role our model may take in the future as a rule-out test for omitting SB and not as a rule-in test. The model also has a higher clinical benefit in the lower probability thresholds compared to the current standard of performing SB for everyone. Our results show that relying on the model would lower the number of biopsy cores taken, potentially lowering the complication rate, while maintaining the high csPC detection rates. To our knowledge, this is the first prediction model developed for this outcome. We also found that prostate volume, PSA, age, and PSAD were highly influential on the model’s prediction. All these features have threshold values that govern their effect on the model. High prostate volume and PSAD values increase the probability of SBD, while lower values decrease it. PSA and age have an inverse relationship with their effect on SBD probability. These threshold values may help the clinician in choosing patients with high SBD probability.

In the present study, we used a modern machine learning algorithm, XGBoost, a robust algorithm for clinical risk prediction, which has been successfully applied across multiple medical domains [19,20,21,22]. Although generalized linear models have been commonly used for outcome prediction in medicine, their capacity to capture complex patterns is limited. Machine learning methods can address this limitation by automatically identifying and modeling relevant variables and nonlinear relationships. For example, Liu et al. compared XGBoost and logistic regression for the prediction of sepsis in severe burn patients. The authors showed that the XGBoost model was superior to the LR model in predictive efficacy [23]. Abraham et al. trained XGBoost and LR models on 1296 patients with 24 h urine and electronic health record (EHR) data to predict kidney stone composition. For binary discrimination of calcium versus non-calcium stones, XGBoost achieved higher accuracy than LR (91% vs. 71%) [24].

Our study has several limitations. Firstly, the retrospective nature of the study may introduce unknown confounders not accounted for by our model. For example, prior biopsy history was not collected, but it may influence the results of the model’s prediction. However, we used all known pre-biopsy available features that are in clinical use today. Secondly, our SBD definition included patients with non-PC in the SB and no PC in the TB, which is considered overdiagnosis today. Our results should be regarded as hypothesis-generating. Prospective validation of our model needs to be performed before entering clinical use. Thirdly, the use of advanced pre-biopsy information from radiologic and genomic big data may increase the accuracy of a prediction model, which will be evaluated in future works. Finally, we performed multiple imputations on the entire dataset and not the training set only. This may introduce overly optimistic test performance; however, because the proportion of missing predictor data was small in our dataset, this bias will be minimal.

## 5. Conclusions

Currently, the standard of care is to perform both SB and TB during FB. However, the balance of cancer detection and adverse events should be individualized. We developed a prediction model that, after external validation, may assist the clinician in choosing when to omit SB during FB.

Our prediction model is able to accurately predict which patients must undergo SB with TB and has the potential to help in the decision whether to perform SB, thus, may lower the adverse event rate while keeping a high detection rate.

## Figures and Tables

**Table 1:** Clinical features of patients included in the study by dominance status. SBD patients were older and had smaller prostates compared to TBD patients. All other features were not significantly different between the dominance groups.

Characteristic	TB Dominant, N = 447	SB Dominant, N = 82	*p*-Value
**Age [y]**	74 (68, 80)	66 (61, 71)	<0.001
**PSA**	6.2 (4.8, 9.0)	6.3 (4.8, 9.4)	0.8
**Prostate Volume [cc]**	48 (36, 63)	58 (43, 75)	0.002
**No of MRI lesions**	2.00 (1.00, 2.00)	2.00 (1.00, 2.00)	>0.9
**Max PIRADS in ROI**	4.00 (3.00, 4.00)	4.00 (3.00, 4.00)	0.4
**Histologic region of maximal GG [PZ/other]**			>0.9
Other	100 (22%)	18 (22%)	
PZ	347 (78%)	64 (78%)	
**PSAD**	0.12 (0.08, 0.17)	0.13 (0.07, 0.21)	>0.9
**Clinical T stage**			0.6
1	314 (70%)	62 (76%)	
2	70 (16%)	8 (9.8%)	
3	63 (14%)	12 (15%)	
**Gleason group in TB**			<0.001
No malignancy	147 (33%)	56 (68%)	
1	116 (26%)	15 (18%)	
2	94 (21%)	7 (8.5%)	
3	56 (13%)	0 (0%)	
4	11 (2.5%)	4 (4.9%)	
5	23 (5.1%)	0 (0%)	
**Gleason group in SB**			<0.001
No malignancy	193 (43%)	0 (0%)	
1	119 (27%)	43 (52%)	
2	73 (16%)	17 (21%)	
3	38 (8.5%)	13 (16%)	
4	5 (1.1%)	3 (3.7%)	
5	19 (4.3%)	6 (7.3%)	

## Data Availability

The dataset will be available on request from the corresponding author.

## Supplementary Materials

The following supporting information can be downloaded at: https://www.mdpi.com/article/10.3390/cancers18030517/s1, Table S1: Clinical features of patients included in the training and test sets.

## Abbreviations

The following abbreviations are used in this manuscript:

PC	Prostate cancer
FB	Fusion biopsy
TB	Targeted biopsy
SB	Systematic biopsy
csPC	Clinically significant prostate cancer
ROI	Region of interest
GG	Grade group
GS	Gleason score
PSA	Prostate-specific antigen
PSAD	PSA density
SBD	SB dominant
XG	Extreme gradient boosting
ROC	Receiver–operating characteristic
SHAP	Shapley Additive Explanation

## References

[1] Siegel R.L., Giaquinto A.N., Jemal A. (2024). Cancer statistics, 2024. CA Cancer J. Clin..

[2] Cornford P., van den Bergh R.C.N., Briers E., Van den Broeck T., Brunckhorst O., Darraugh J., Eberli D., De Meerleer G., De Santis M., Farolfi A. (2024). EAU-EANM-ESTRO-ESUR-ISUP-SIOG Guidelines on Prostate Cancer-2024 Update. Part I: Screening, Diagnosis, and Local Treatment with Curative Intent. Eur. Urol..

[3] Mottet N., van den Bergh R.C.N., Briers E., Van den Broeck T., Cumberbatch M.G., De Santis M., Fanti S., Fossati N., Gandaglia G., Gillessen S. (2021). EAU-EANM-ESTRO-ESUR-SIOG Guidelines on Prostate Cancer-2020 Update. Part 1: Screening, Diagnosis, and Local Treatment with Curative Intent. Eur. Urol..

[4] Drost F.H., Osses D.F., Nieboer D., Steyerberg E.W., Bangma C.H., Roobol M.J., Schoots I.G. (2019). Prostate MRI, with or without MRI-targeted biopsy, and systematic biopsy for detecting prostate cancer. Cochrane Database Syst. Rev..

[5] Rouvière O., Puech P., Renard-Penna R., Claudon M., Roy C., Mège-Lechevallier F., Decaussin-Petrucci M., Dubreuil-Chambardel M., Magaud L., Remontet L. (2019). Use of prostate systematic and targeted biopsy on the basis of multiparametric MRI in biopsy-naive patients (MRI-FIRST): A prospective, multicentre, paired diagnostic study. Lancet Oncol..

[6] Wegelin O., Exterkate L., van der Leest M., Kelder J.C., Bosch J., Barentsz J.O., Somford D.M., van Melick H.H.E. (2019). Complications and Adverse Events of Three Magnetic Resonance Imaging-based Target Biopsy Techniques in the Diagnosis of Prostate Cancer Among Men with Prior Negative Biopsies: Results from the FUTURE Trial, a Multicentre Randomised Controlled Trial. Eur. Urol. Oncol..

[7] Hou N., Li M., He L., Xie B., Wang L., Zhang R., Yu Y., Sun X., Pan Z., Wang K. (2020). Predicting 30-days mortality for MIMIC-III patients with sepsis-3: A machine learning approach using XGboost. J. Transl. Med..

[8] Ogunleye A., Wang Q.G. (2020). XGBoost Model for Chronic Kidney Disease Diagnosis. IEEE/ACM Trans. Comput Biol. Bioinform..

[9] Eng J. (2003). Sample size estimation: How many individuals should be studied?. Radiology.

[10] Lundberg S.M., Lee S.-I. (2017). A unified approach to interpreting model predictions. Adv. Neural Inf. Process. Syst..

[11] Yang W., Jiang J., Schnellinger E.M., Kimmel S.E., Guo W. (2022). Modified Brier score for evaluating prediction accuracy for binary outcomes. Stat. Methods Med. Res..

[12] Van Calster B., Wynants L., Verbeek J.F.M., Verbakel J.Y., Christodoulou E., Vickers A.J., Roobol M.J., Steyerberg E.W. (2018). Reporting and Interpreting Decision Curve Analysis: A Guide for Investigators. Eur. Urol..

[13] Azur M.J., Stuart E.A., Frangakis C., Leaf P.J. (2011). Multiple imputation by chained equations: What is it and how does it work?. Int. J. Methods Psychiatr. Res..

[14] Benjamini Y., Cohen R. (2017). Weighted false discovery rate controlling procedures for clinical trials. Biostatistics.

[15] Collins G.S., Reitsma J.B., Altman D.G., Moons K.G. (2015). Transparent Reporting of a multivariable prediction model for Individual Prognosis or Diagnosis (TRIPOD): The TRIPOD statement. Ann. Intern. Med..

[16] Borghesi M., Ahmed H., Nam R., Schaeffer E., Schiavina R., Taneja S., Weidner W., Loeb S. (2017). Complications After Systematic, Random, and Image-guided Prostate Biopsy. Eur. Urol..

[17] Pepe P., Aragona F. (2014). Prostate biopsy: Results and advantages of the transperineal approach-twenty-year experience of a single center. World J. Urol..

[18] Ahdoot M., Wilbur A.R., Reese S.E., Lebastchi A.H., Mehralivand S., Gomella P.T., Bloom J., Gurram S., Siddiqui M., Pinsky P. (2020). MRI-Targeted, Systematic, and Combined Biopsy for Prostate Cancer Diagnosis. N. Engl. J. Med..

[19] Ellmann S., von Rohr F., Komina S., Bayerl N., Amann K., Polifka I., Hartmann A., Sikic D., Wullich B., Uder M. (2025). Tumor grade-titude: XGBoost radiomics paves the way for RCC classification. Eur. J. Radiol..

[20] Liang D., Wang L., Zhong P., Lin J., Chen L., Chen Q., Liu S., Luo Z., Ke C., Lai Y. (2025). Perspective: Global Burden of Iodine Deficiency: Insights and Projections to 2050 Using XGBoost and SHAP. Adv. Nutr..

[21] Shi J., Chen L., Yuan X., Yang J., Xu Y., Shen L., Huang Y., Wang B., Yu F. (2025). A potential XGBoost Diagnostic Score for Staphylococcus aureus bloodstream infection. Front. Immunol..

[22] Wang X., Zhou L., He M., Peng Z., Zhang J. (2025). XGboost with multi-feature fusion for hemodynamic state prediction. Neuroscience.

[23] Zhang Y., Wang Y., Xu J., Zhu B., Chen X., Ding X., Li Y. (2021). Comparison of Prediction Models for Acute Kidney Injury Among Patients with Hepatobiliary Malignancies Based on XGBoost and LASSO-Logistic Algorithms. Int. J. Gen. Med..

[24] Abraham A., Kavoussi N.L., Sui W., Bejan C., Capra J.A., Hsi R. (2022). Machine Learning Prediction of Kidney Stone Composition Using Electronic Health Record-Derived Features. J. Endourol..

