# Supplementary material for: Is Systematic Biopsy Mandatory in All MRI-Guided Fusion Prostate Biopsies? A Machine Learning Prediction Model
Source: Cancers (Basel). 2026 Feb 4;18(3):517. doi: 10.3390/cancers18030517 (PMC12896627; doi:10.3390/cancers18030517)
Supplement: Supplementary file 1 [file cancers-18-00517-s001.zip › cancers-4058215-supplementary.pdf]

Table S1: Clinical features of patients included in the training and test sets.

	Training Set, N = 369	Test set, N = 160	q-value
**AGE**	67 (62,73)	67 (62,72)	>0.9
**PSA**	6.2 (4.8,9.0)	6.5 (4.7,9.4)	>0.9
**Prostate Volume[cc]**	57 (43,75)	56 (37,72)	>0.9
**No of MRI lesions**	2.00 (1.00,2.00)	2.00 (1.00,2.00)	>0.9
**Max PIRADS in ROI**	4.00 (3.00,4.00)	4.00 (3.00,4.00)	>0.9
**Histologic region of maximal GG [PZ/other]**			>0.9
Other	79 (21%)	39 (24%)	
PZ	290 (79%)	121 (76%)	
**PSAD**	0.12 (0.08,0.17)	0.13 (0.08,0.20)	>0.9
**Clinical T stage**			>0.9
1	258 (70%)	118 (74%)	
2	56 (15%)	22 (14%)	
3	55 (15%)	20 (13%)	
**Highest risk PC**			>0.9
TBD	312 (85%)	135 (84%)	
SBD	57 (15%)	25 (16%)	
**Gleason group in TB**			>0.9
No malignancy	141 (38%)	62 (39%)	
1	89 (24%)	42 (26%)	
2	79 (21%)	22 (14%)	
3	32 (8.7%)	24 (15%)	
4	12 (3.3%)	3 (1.9%)	
5	16 (4.3%)	7 (4.4%)	
**Gleason group in SB**			>0.9
No malignancy	135 (37%)	58 (36%)	
1	108 (29%)	54 (34%)	
2	68 (18%)	22 (14%)	
3	33 (8.9%)	18 (11%)	
4	6 (1.6%)	2 (1.3%)	
5	19 (5.1%)	6 (3.8%)	
